# Clinical Review, and Hospital Reports

**Published:** 1843-10-01

**Authors:** 


### Clinical Review.

---

### Clinical Lectures on the Theory and Medical Treatment of Insanity. By Alexander John Sutherland, M.D.

These excellent Lectures, which were delivered at St. Luke's Hospital, in the early part of May, are published in the *Medical Gazette*. We shall select a few points for extract.

*Hallucinations and Illusions.*—These terms have often been misunderstood. Hallucination is a creation from within, illusion is a mistake from without: the mirage is an example of an illusion which the thirsty traveller of the desert is taught to correct. Supposed visions are examples of hallucinations; both the one and the other are compatible with perfect sanity; for example, our Saxon ancestors believed that they were pursued by the fauns, forest-fiends, &c., which haunted the abodes of their fathers in the wilds of Germany. We call this superstition, not insanity. Visions of imagination have also appeared to all classes of persons; young and old, saints and sinners. Such examples are often met with in cases of aberration of intellect; the nerves of hearing and of sight are most commonly the seat of injury; commands issuing from supernatural voices, warnings from deceased friends, persecutions from ideal enemies, visions of angels or of devils, frequently embarrass the bewildered imagination; the nerves of touch are certainly less commonly found disordered than the rest. In some cases, hallucination of one sense, and illusion of another exist together.

Examples of hallucination of smell are not very uncommon, especially in the case of those who imagine that they suffer the tortures of the condemned; these delusions, also, often accompany alterations of the nerve of taste. False perception of taste, unconnected with a foul tongue, and derangement of the stomach and bowels, are rare. A patient imagined she had murdered a child; she refused her food because she thought it was the flesh of the victim she had slain, and would not drink, because every thing she swallowed tasted, she said, like human blood. The tongue was not loaded, the bowels were not costive; the false perception, therefore, must be accounted for either by supposing, with Esquirol, that it was created by the imagination, or by concluding, with Foville, that it was due to disease at the origin of the nerve in the brain, not to its termination in the mouth.

*Hallucinations of the Nerves of Touch* are the most rarely met with: under this head the following instances may be placed.

A young officer imagined that by touching people, he had the power of magnetizing and attracting them. Another patient imagined that her head had been cut off, and that it was rolling about the room like a ball. The story of the turned head, so well told by the able author of the "Diary of a Physician," is not mere fiction. Dr. Sutherland has met with cases very similar to it. One man thought that his head was set on the wrong way, (as the poor person who was saved from the guillotine). A patient, now in St. Luke's, fancies that all her bones are turned round.

*Illusions from Morbid Impressions on the Nerves.*—Many examples of these are given. It is remarkable how readily the patient seizes upon some slight impression upon any of the senses, and converts it into something connected with his delusion. Dr. Sutherland happened to prescribe the compound galbanum pill for an old sportsman, who was labouring under insanity with hypochondriasis: he thought, from the smell of the assafœtida, that he had been metamorphosed into a fox, and that he was to be turned out the next morning before the hounds. A gentleman, labouring under a religious delusion happened one evening to be in a large room lighted up by a single lamp, which occasioned a flickering light to appear on the cieling. The gentleman immediately declared that the roof had opened, and that St. John, surrounded by a halo of glory, was bidding him be of good cheer. An Irishwoman in St. Luke's, a short time ago, after listening to the sounds of the clock for some time, stopped it because she thought it was calling her names.

Dr. Sutherland remarks, that it is of great importance in practice to attend to these altered perceptions; if you set them down as the mere effects of imagination, and do not stop to inquire whence they arise, and what is the probable cause of their existence, you will undoubtedly treat the disease very badly; and your attention may at last be called to the symptom when medicine is of no avail. "Patients either with or without the sensation of pain about the præcordia, imagine that they are possessed with devils, or have live animals in their stomach: I recommend you never to overlook such symptoms. Some madmen eat their food voraciously, and without even attempting to masticate it. A patient in St. Luke's, who was in the habit of doing this, had had a severe fall upon the back part of his neck: his friends said, he never knew when he had enough to eat: it is not impossible that the eighth pair of nerves might have been injured by the fall. There are those who eat their own flesh, and other matters which I need not mention here. This is always a very bad symptom. There are others who refuse food, and it has been too often the habit to order them to be fed with the stomach-pump, without taking into consideration the state of the bodily symptoms. I am confident that the majority of patients who refuse to eat, do so because there is irritation of the stomach and primæ viæ: if you order an emetic or a purgative, you will generally find this symptom disappear.

Esquirol never saw any dangerous consequences ensue in mania from obstinate refusal of food: in monomania, however, the case is otherwise: patients who have tried every other means to destroy themselves, will sometimes endeavour to starve themselves to death, and all our art is called into requisition to avoid such a catastrophe."

*Capability of the Insane to bear Heat and Cold.*—This, as well as the power of doing without sleep, Dr. Sutherland thinks has been over-stated. It is very true, that some patients can expose themselves to the rays of the sun, and go almost naked in the coldest winter, with impunity; but this insanity is not only the result of the abstraction of mind, it is owing also to the benumbing effect of the disease upon sensibility generally. If you do not protect these patients against extreme cold, or intense heat, you will soon find them suffering, on the one hand, from mortification of the feet, and, on the other, from repeated exacerbations of furor, which too probably will end in dementia: and if you do not take proper precaution, that your patient be soothed by refreshing sleep, the exhaustion subsequent to his long watchfulness will be a great hindrance to his recovery.

*Alterations of Motion.*—The motive power may be increased or diminished in intensity. It is sometimes increased to a surprising extent during the maniacal paroxysms, and in catalepsy. In some rare cases, the patient will turn neither to the right hand nor to the left, but runs straight on, impelled by some irresistible impulse, till stopped either by exhaustion, or some obstacle obstructing his course. One of these cases, was a young lady under Dr. Sutherland's care, whose insanity was manifested, in the first instance, by her getting out of bed in her night-dress, and going in a direct line over hedges or anything which came in her way, till she was at length, with difficulty, extricated from a pond. A female patient, when first admitted into St. Luke's, would get off her chair, and walk round two or three times in a circle, the left side being always turned towards the centre. These cases are interesting, when we reflect that similar results followed the removal of the corpora striata, and lesion to the medulla oblongata, in M. Magendie's experiments.

When the motor nerves are affected in cases of insanity, it is always a bad sign. Esquirol says, that insanity when complicated with lesions of motion, or with epilepsy, is never recovered from. Dr. Sutherland, however, considers that it makes an essential difference if the lesion of motion has preceded, not followed, insanity: he has seen insane persons completely recover who have been epileptic, provided the fits came on first, the madness afterwards; and the same with paralysis.

"Ptosis of the eyelid, and strabismus, are bad symptoms, more especially if either is permanent. A synchronous action of the pupil, dilatation of one iris, and contraction of the other, are also bad signs; but if the sphincters be involved, the patient is not likely to recover, and if the muscles of deglutition participate in the mischief, the case is hopeless."

If general paralysis occur, the case may be considered incurable. It is of great consequence to accustom the ear to the speech of those suffering under this form of madness; the first sentence the patient utters will generally enable you to detect the disease; there is a thickness, a hesitation in speaking, very like the speech of the drunkard. Patients have a difficulty in pronouncing the letter R; if you make them say the word February, they will generally fail. There is very frequently a general tremor of the muscles; occasionally this tremor may be observed in the upper lip alone, the muscles in the other parts of the body not being affected. It is not the power so much as the general precision of the voluntary motions which is lost. If a patient, for instance, attempts to write, he will scarcely be able to hold a pen; or if he walks, there is an irregular gait, he leans over to one side, and drags one of his legs. The last stage of the complaint is accompanied by sloughing sores on the sacrum, incontinence of urine and fæces, death by coma, convulsions, or epilepsy.

The delusions which accompany general paralysis are very frequently those of great magnificence. The patients imagine that they have millions of money; that they are about to pay off the national debt; they often also think themselves happier and stronger than they were before. Soon, however, a change takes place, the faculties of the mind fail, and hopeless fatuity succeeds.

With regard to the pathology of this form of general paralysis; Esquirol says that it is often a symptom of chronic inflammation of the meninges. M. Calmeil considers that it is due to disease of the cortical substance. M. Foville believes it to depend upon alteration of the fibrous structure; he says that adhesions exist between the planes, which are easily separated in a healthy person, but cannot be in many of those of the insane; every effort you make to do so only tears them. Dr. Sutherland, however, believes that this last hypothesis is not correct, at least in all cases, as he has seen the fibres quite as disunited in general paralysis as in health. The appearances most often found are, perhaps, considerable injection of the medulla oblongata; effusion of fluid in the subarachnoid cellular tissue and in the ventricles; the pia mater thickened; the substance of the brain infiltrated, sometimes soft, sometimes tough and elastic, with the fibres well marked, as if the brain had been for some time steeped in alcohol. The disease, it appears, does not always commence in the brain and thence spread itself to the spinal marrow; it sometimes commences in the medulla oblongata and radiates to the hemispheres.

*State of the Pulse.*—Many authors assert that the pulse is not affected in cases of insanity; this, however, does not accord with Dr. Sutherland's experience, as will appear from the state of the pulse as recorded in 60 patients admitted into St. Luke's.

| | On admission. | On discharge. |
|---|---|---|
| | from 40 to 50 | in 1 case. |
| In 5 cases | from 60 to 70 | in 2 cases. |
| | from 70 to 80 | in 1 case. |
| In 6 cases | from 80 to 90 | in 21 cases. |
| In 4 cases | from 90 to 100 | in 3 cases. |
| In 34 cases | from 100 to 120 | in 20 cases. |
| In 1 case | above 120 | in 2 cases. |

The pulse was slower than on admission when the patient left the hospital in 31 cases, quicker in 12 cases, equal in 7 cases. Hypertrophy of the heart is not very uncommon in this disease.

*Is insanity an inflammatory disease?*—It very seldom happens that we find in insanity acute inflammation. If such symptoms were present it would be impossible to distinguish phrenitis from insanity. If fever is present, which is not very generally the case, it follows the delirium in insanity, whilst it precedes it in phrenitis. In delirium, the mind is occupied solely with the past, in madness with the past and present also. In phrenitis, the blood presents the buffy coat, in mania it does so very seldom. In post-mortem examinations of the insane, we meet with the results of congestion or of chronic inflammation, seldom those of acute inflammation. This is a point of practical import, "for if you are called in to see a patient in a paroxysm of mania, and have read that the disease owes its origin to inflammation, you would be doing very wrong if you were to bleed him freely. I believe that every variety of congestion exists in insanity, whether active, passive, or mechanical. The blood is either in excess, or deficient in quantity, and the circulating fluid acts sometimes as a poison to the brain. The reason why we find variation, I had almost said contradictions, in the mode of treating the same case of insanity is, because one thinks only of inflammation, and bleeds, another considers the symptoms due to irritation, and employs opiates or stimulants."

It is frequently of service to place the insane patient in a recumbent posture, in order to see whether the symptoms are aggravated or relieved by the gravitation of the blood towards the head, as this serves as a useful guide in the employment of remedies.

Passive congestions, the blood appearing to stagnate in the capillaries, not unfrequently occur in cases of insanity. Patients in this condition are generally weak and sluggish in their movements; little animal heat is evolved; the skin loses much of its sensibility; the pulse is so weak as to be scarcely felt. The lips of the patient are often blue, on assuming the recumbent position, they regain their natural colour. Occasionally sudden congestion occurs, ending frequently in apoplexy. Congestion of the liver is not uncommon in cases of insanity; organic disease of this viscus, however, is rare.

Sleeplessness is a very common symptom in the early period of insanity. Dr. Bright attributes the frequent attacks which epileptics have in the night to slight congestion, which he says always accompanies sleep. This is the reason that the insane so often do not enjoy this temporary release from care. Unless there be some bodily disease, as fever, present, a patient should never be allowed to remain in bed during the day, as the recumbent position will of course favour the congestion.

*Complications of Insanity.*—" Although there is no disease with which insanity may not be complicated, yet there are some more commonly met with in conjunction with it, than others: *e. g.* scrofula, phthisis, inflammation of the lungs, disease of the heart, congestion of the liver, diseased kidneys, inflammation of the mucous lining-membrane of the intestines, diarrhœa, erysipelas, epilepsy, apoplexy. Sometimes an attack of diarrhœa, of phthisis, or of fever, will put a stop to the attack of insanity; sometimes the attack will be merely suspended. The approach of the catamenia is always a critical period with our female patients: the disease is then often aggravated, and paroxysms of fever occur: the return of the monthly period sometimes gives an intermitting character to the disorder, and the hopes of the friends and of the physician are alternately raised and depressed. It is not uncommon to meet with insanity manifesting itself in one member of a family, while nervous disorders akin to it develop themselves in the rest. This takes place, also, occasionally, with respect to other diseases, which are not of a nervous type, *e. g.* scrofula and phthisis." '

*Post-mortem Examinations.*—Dr. Sutherland remarks that nothing is ever found in the brain of the madman which can be said to be characteristic of the disease. Perhaps the more general appearances are these:—The dura mater adheres more or less firmly to the pia mater. The vessels and sinuses of the dura mater are sometimes gorged with blood. The arteria meningea media is frequently injected to its most minute ramifications. The membrane itself is sometimes also much thicker than natural; small deposits of bone are also occasionally found, particularly on the falx. The glandulæ pacchioni are not more numerous in insanity than in other diseases. The arachnoid membrane is very frequently found opaque and thickened; the opacity being sometimes general, sometimes partial. It is more frequently met with on the surface of the hemispheres than at the base of the brain. M. Foville considers the opacity and thickening of the arachnoid, and its adhesions with the pia mater, not to be the simple effects of inflammation, but of a deposit of albuminous layers.

The arachnoid is sometimes perfectly dry; its surfaces are not lubricated by the smallest quantity of serum. In general however, there is much effusion of clear transparent fluid into its cellular tissue, it is not often that this fluid is coloured. This appearance must not be taken as an unequivocal proof of inflammation of the arachnoid: for it may be caused by congestion of the veins, or be the result of poverty of blood.

Dr. Sutherland thinks that this effusion is often the consequence of atrophy of the convolutions, as he has observed, in some cases where the atrophy has been very limited in extent, that serum has collected here under the arachnoid, which has shown no signs of inflammation: upon one or two occasions this effusion of fluid has been so circumscribed by the membrane as to be at first mistaken for a transparent cyst.

The pia mater is sometimes found adherent to the arachnoid on the one side, to the convolutions on the other. It is sometimes much thickened, and in recent cases generally injected. There is great variation in the quantity of blood, and in the state of the capillary vessels.

*Alterations in the Cortical Structure.*—In acute mania, the convolutions have a tendency to hypertrophy, in chronic mania to atrophy. In acute cases the pia mater is seldom found adhering to the surface of the hemispheres; the convolutions are well developed; when cut there is as well-defined a separation between the cineritious and fibrous structure as in a healthy brain; the white bands may be traced in every part running parallel to the external and internal surface; but the cortical structure is much injected, and frequently presents a marbled appearance.

The chronic alterations are more marked than those of the acute. The pia mater frequently adheres to its surface; and a layer of softened gray matter is sometimes separated in the attempt to peel it off. The diminution of bulk takes place both on the surface and in the centre of the convolutions, so that wide sulci are left between the folds, and a very thin layer is spread over the fibrous structure: the white bands have either disappeared, or the colour of the ganglion globules is so faded that they cannot be distinguished. The external layer of the cortical structure is sometimes firm while the internal layer is soft.

*Alterations of the Fibrous Structure.*—On slicing the brain numerous bleeding punctæ are frequently seen. This varies in intensity from the few specks seen in healthy brains to patches of extravasated blood. Too much stress, however, as Dr. Seymour observes, must not be laid on this appearance. Sometimes the brain is extremely firm, appearing like hard albumen; at other times the brain is remarkably soft. Ramollissement is not very uncommonly met with. The ventricles are frequently distended with fluid, and their walls found gaping and inelastic.

Dr. Sutherland in his analysis of these post-mortem observations draws attention—1st, to the many different morbid appearances which accompany the disease; 2ndly, to the many similar appearances which give rise to its different species, and are found where insanity does not exist.

"Now although we may look to the cortical substance as giving us the most conclusive evidence of variations from healthy structure, yet we are far from being able to say in what the first departure from such healthy structure consists. Do we call it congestion? This is the mere effect of something which has caused previous irritation. We know well how different in their nature the causes are which produce congestion—whether passion, over-exertion of intellect, diseases of the heart and lungs, stomach and intestines, narcotic poisons, &c. It would appear, then, that beyond what morbid anatomy teaches us, there is some change in the brain which, influenced by individual susceptibility, is the primary cause of the various phenomena which we recognize in madness. In this, as in every other disease, morbid anatomy alone cannot guide us in establishing just views of pathology."

*Medical Treatment.*—Dr. Sutherland confines his observations in these lectures to the medical treatment of insanity, as it is a subject which has been, from various causes, much neglected. Of course it is necessary to be fully cognizant of disease in the sane patient, in order to detect it when masked by insanity. It requires great observation and attention to be able to separate the real from the imagined pains of the lunatic; the previous diseases, habits or occupation of the patient may tend to throw some light upon the method of treatment.

Dr. Sutherland advises a systematic arrangement in each case. "Observe the state of the pulse and the skin; feel the head; see whether it is hot all over, or in one part only; whether the extremities are cold; whether the tongue is loaded and dry; whether the bowels are open, the urine free, and, if the patient be a female, whether the catamenia are regular. Next observe the breathing, and the action of the heart; pass your hand over the right hypochondrium, and feel whether the liver is enlarged, or whether the abdomen is distended with flatus; and whether there be tenderness about the præcordia. Examine also the beating of the carotids and temporal arteries. When the patient has his head back, and his neckcloth unfastened, you may perhaps see a scar on the throat, which will let you into a secret. The expression of countenance, the complexion, the colour of the conjunctivæ, and the action of the iris, must all be carefully noted. When you are observing the eye, ask the patient whether he sees moats floating before him; and this may lead him to speak of his illusions or hallucinations. When you have exhausted these, ask about his sense of smelling; you may discover, perhaps, that he smells brimstone, and imagines himself lost to all eternity. Now inquire whether his taste is altered; if it be, he may fancy that his friends have conspired to poison him. Then pay attention to the sense of hearing; and lastly, to that of touch. You will now be prepared to draw him into conversation, in order to ascertain whether his emotions are changed, or whether one or more of the faculties of the mind be disordered."

*Purgatives.*—The use of these medicines is of great importance, from the great frequency of constipation in insanity, and from the fact that some cases recover after a severe diarrhœa. The preparations of mercury are very useful, not only for their purgative effects, for stimulating the liver and relieving it of the congestion so often found in recent cases, but they are of great service in equalizing the circulation in the capillaries; thus in those cases where, from the heat of head, the injected conjunctivæ, and other symptoms, we have to fear congestion of the cerebral vessels, their use is manifestly indicated. In cases where there is reason to suspect incipient paralysis, or even where the disease has begun to develop itself, Dr. Sutherland has derived much service from the liquor hydrargyri bichloridi, given in ʒss. to ʒij. doses two or three times daily, with occasional purges. The importance of continuing this medicine for a considerable time has not been sufficiently insisted upon in such cases: it is long, indeed, before the specific effects are felt in the constitution; and it does not seem necessary that these effects should be produced in order that the good results should follow.

In the first stage of mania, when there is heat of skin and of the head, it is as well to give a full dose of calomel, repeated for two or three nights in succession, according to circumstances. Or blue-pill may be given with the extract of colchicum, to procure an abundant discharge of bile. These will be of great service if the tongue is loaded and the conjunctivæ yellow. If the patient refuse medicine the endermic method may be employed.

"If the patient has recovered from the acute stage of mania, and the evacuations are still unhealthy, and the action of the liver sluggish, with much general debility, you will find it of use to give the nitro-muriatic acid in a light bitter infusion. The extract of taraxacum is likewise a drug which has its use, both as acting on the liver, and for its diuretic effects. It may be combined with the aromatic spirit of ammonia, in cases where there exists depression of spirits and flatulence. Among the purgatives derived from the vegetable kingdom, jalap and rhubarb are most in vogue. The house-medicine at Bethlehem hospital is calomel and rhubarb; that of St. Luke's, the former with jalap. Where you wish to have a copious discharge from the exhalant vessels, and to draw upon the excretory ducts of the mucous glands of the intestines, and to increase the secretion of the kidneys, jalap is a most serviceable remedy, and one which you may, generally speaking, safely employ. If you wish a more powerful drastic purgative, you must have recourse to elaterium; but this will seldom be either necessary or safe."

Two cathartics Dr. Sutherland is in the habit of giving to the hospital patients: one, a purgative with some vegetable bitter infusion, as in the mist. gentianæ comp. of the Pharmacopœia, for those whose muscles are relaxed, and who might not be able to bear an active purgative. The other is the croton oil, for those patients who refuse medicine, and where it is essential to promote regular action of the bowels.

*Emetics.*—These were at one time much recommended, Dr. Sutherland, however, does not often employ them in insanity for two reasons. First, because where there is a tendency to congestion in the vessels of the head, they are known to increase it; secondly, because the nerves of the stomach in insanity, as those of the intestines, are frequently less sensitive to impressions than in a state of health, owing to the disease of the brain; and it is necessary to give a large dose before the stomach will act. Nauseating doses of these remedies are valuable. A fourth of a grain of tartar emetic may be given every four hours, or at the commencement of the paroxysm of furor; this controls powerfully the action of the heart and arteries. In many cases it acts like a charm, subduing the excitement and violence of the patient, and sometimes an alteration of the symptoms for the better commences with its administration. If, however, the doses are large and repeated, great prostration of strength may be produced.

*Narcotics.*—Much difference of opinion exists with regard to the benefit to be derived from their administration. Dr. Sutherland's experience leads him to the conclusion, that opiates are of essential service in those cases of insanity which border closely upon delirium tremens; in cases of puerperal mania; in the first breaking out of an attack of madness, before congestion has taken place; in cases where there is great nervous irritability, from poverty of blood; and in cases of cachexia from starvation, and other causes: they are contra-indicated wherever there is the least sign of general paralysis or congestion about the head.

Hyoscyamus and conium may be employed as well as opium and morphia. Hyoscyamus often agrees better with the stomach, and it does not constipate the bowels; it also increases the secretion of the kidneys and of the skin. It is often serviceable, combined with tartar emetic, in paroxysms of furor.

Combined with camphor, opium allays the irritability of those suffering under madness, either accompanied with some degree of delirium tremens, or preceded by it. Stramonium, belladonna, and aconite are not, in general, of very much importance.

Hydrocyanic acid is useful as a sedative in those cases where there is pain and a sense of weight about the præcordia. If acid eructations are present, it may be combined with soda, or, if there be much action of the heart and arteries, with digitalis.

*Diuretics.*—The urine of insane patients is very often, at the commencement of the attack of mania, scanty and high-coloured, with a lateritious sediment. Sometimes no water is secreted for a day, sometimes for two days. In these cases nitric æther, with nitrate of potash, infusion of digitalis, or the compound decoction of scoparius may be given with advantage. The more powerful diuretics are seldom of much service; even where you suspect effusion, or in incipient paralysis, the bichloride of mercury is generally to be preferred to cantharides.

*Tonics.*—Since the insane have been better fed, there have been, on an average, more recoveries and fewer deaths. Even in acute mania it is necessary to have reference to the future condition in our treatment. Light bitters, mineral tonics, the preparations of iron, the sulphate of zinc, and the salts of copper, are often valuable medicines in nervous disorders.

*Blood-letting.*—This practice fortunately does not prevail in England as it formerly did. Even in acute mania with symptoms of plethora, local abstraction of blood by means of leeches is a much more safe means of proceeding.

*Counter-irritants.*—These are much employed in the treatment of insanity. They should not, however, be used in the acute stage of mania, certainly not till the heat of skin and general irritation from the loaded vessels have subsided. The acetum instead of the emplastrum lyttæ ought generally to be employed.

In some cases of insanity which run their course sluggishly, or where there is a healed ulcer, or a suppressed discharge, setons are of great service. Esquirol says they act as if by enchantment. The ung. antim. pot. tart. proves beneficial in some cases of suppressed eruptions, and as a counter-irritant.

Strychnine has proved serviceable in catalepsy; it is also useful in cases of insanity accompanied with paralysis. Where patients have illusions of hearing, cotton, on which laudanum has been dropped, may be put in their ears: it is also useful to have the ears syringed, or to place small blisters behind each ear, to divert the attention of the patient from the noises and whisperings on which his imagination dwells.

The function of the skin is often disordered in insanity, to remedy which state baths of every description have been, very properly, recommended. The tepid bath is of great service in subduing irritability and excitement. It is sometimes necessary that the patient should remain in it for an hour and a half or two hours. Ice or cold lotions to the head should be applied at the same time: it may be necessary to repeat it every day, sometimes twice daily, till some effect is produced.

If the irritation is not subdued, a blister may be applied to the nape of the neck, immediately the patient leaves the bath. In acute dementia, much benefit may be derived from the douche, but this is a remedy which requires caution, and is not by any means to be ordered for those patients who are liable to congestion of the head, or have any tendency to paralysis.

The shower bath, with antispasmodics, is a valuable means of subduing the symptoms of madness with hysteria and hypochondriasis.

When a patient has passed into what is termed the chronic stage of the complaint it by no means follows that nothing is to be done for him; something can be effected by medicine even here, in shortening the paroxysms of furor, in procuring sleep for the restless, and improving the general health of the debilitated.

---

### ST. GEORGE'S HOSPITAL.

### Report of Cases occurring in the Practice of Mr. Henry James Johnson, Assistant-Surgeon to the Hospital.

The following are selected from amongst the many of a miscellaneous character, which have been under treatment lately.

#### I. CHRONIC ABSCESS IN THE SUBSTANCE OF THE TONGUE.

*Case.*—Thos. Adams, aged 17, a vendor of cat's-meat, came under my care on the 10th of May of this year.

In the substance of the tongue, on the right side of its median line, and about three quarters of an inch from the tip, is a hard, globular tumor, of about the dimensions of a small marble. There is occasionally some shooting pain in it, and it is rather tender upon pressure. The speech is rendered indistinct by it. No enlargement of lymphatic glands. Face somewhat puffy, but health reported good.

He states that he first perceived the swelling, accidentally, nine months ago. It was then very small, and has increased gradually since. Painless at first, it has become tender within the last three weeks. He can assign no satisfactory cause for it.

The tumor felt as hard as a scirrhous tubercle, but the age and appearance of the lad combined to render that idea improbable. The globular form and firm resistance conveyed to me the impression, that there was either a cyst with thickened walls, or chronic abscess. I therefore introduced the exploring needle, and was gratified to observe a drop or two of distinct, yellow pus escape along the groove. The size of the swelling was little reduced by the evacuation, and it was evident that the parietes of the abscess were so consolidated as to form the bulk of the tumor. I prescribed:—

Pil. hyd. chlor. comp. gr. v, omni nocte;—Dec. sarsæ comp. ℥ij. bis quotidie;—Magnesiæ sulph. ℥ss. p. r. n.

By the 15th of June, one month from the puncture, the tumor was reduced to half its original size, but continued very hard. It was quite unattended with pain, but still occasioned difficulty of articulation.

By the 4th July no trace of the complaint could be detected, but, seemingly from habit, the speech was still thick and indistinct. It may be stated that the gums were never affected, and that the health improved.

A few days after this, he was attacked, without obvious cause, with inflammation of the globus major of the epididymis, on the right side. This speedily subsided under calomel and opium, leeches, saline purgatives, and the application of a belladonna plaister. On the first of August, he was discharged cured.

I apprehend that chronic abscess in the substance of the tongue is rare. Acute inflammation is more common, and cysts are occasionally seen in it. But perhaps the most frequent affections of it are cachectic tubercles and ulcerations on the one hand, and malignant growths, or alterations on the other.

A year or two ago I saw a case which had some little interest attached to it.

### II. TUMOR AT THE SIDE OF THE TONGUE.

A gentleman between forty and fifty years of age, inclined to corpulency, of indolent habits, and rather a bon-vivant, had suffered much, at different times from dyspepsia. Between two and three years ago, he came to me one morning in a state of some alarm, on account of a swelling which he had discovered on the left side of his tongue. It was opposite the first large molar tooth—when the tongue was protruded, it was about the size of a horse-bean, when retracted it nearly disappeared—was of bluish colour, with one or two large veins on it—was little, if at all, painful or tender—and rather occasioned alarm than inconvenience.

On examining it carefully, I could not satisfy myself of the existence of *any* actual tumor, that is of decided increase of bulk, or hardness, or other modification of density. It conveyed to me the impression of mere hypertrophy of the substance of the tongue, palpable only when the organ was thrown into a state of tension by the action of the genio-hyo-glossus and lingualis.

This opinion I ventured to give, and endeavoured to relieve the gentleman's mind by assurances of freedom from danger. But the idea of cancer had taken hold of him, and quite weighed down his spirits.

I prescribed a mild aperient pill to regulate the bowels, and advised the least possible interference with the part. A tooth, which *might* press on it, was, by Mr. Bell's suggestion, taken out.

Actuated by his apprehensions, Mr. B. did not long follow my advice, but applied to an eminent surgeon. This gentleman conceived that the swelling was a cyst, and punctured it with a needle. Nothing, however, but blood issued. It was then touched at stated intervals with caustic, and medicines of an aperient and tonic character given. But, under this treatment, the swelling increased rather than otherwise, the tongue grew foul, and the general health, the effect, probably, of anxiety, deteriorated.

Under these circumstances, Mr. B. consulted Sir Astley Cooper. Sir Astley, after a hurried examination, pronounced the case one of malignant disease, and advised the employment of the ligature without delay. His worst anticipations being now realised, the patient returned to me, and in a state of extreme despondency informed me of Sir Astley Cooper's opinion. I was naturally staggered by this, and made another and a careful examination, without seeing cause to alter my own. I suggested an application to Sir Benjamin Brodie, whose judgment and candour are so worthy of reliance in every case of difficulty. I gave the patient a note to Sir Benjamin, stating what had occurred. He went into the case fully, and gave it as *his* opinion that there was no disease of any consequence, confirming by his high authority the view and the practice that I had adopted.

The relief to the patient's mind was, of course, extreme, but he could not altogether shake off his fears, and, even to this day, he occasionally becomes alarmed. Being let alone, the *tumor* has sensibly declined, and occasions no pain nor annoyance, yet a fulness is still there, or, rather was there, when I saw him last, which is now twelve months ago, but I believe he is quite well in his general health, and thinks or says little of his tongue. I have lately had a very similar case amongst the out-patients of the Hospital. The patient, a girl, was sent to me for collection of fluid in the sub-lingual gland. No such collection existed.

Perhaps the most common affections of the tongue are cachectic tubercle and ulceration on the one hand, and what is sometimes called psoriasis of the tongue, upon the other. A word or two on each may be excused.

### III. CACHECTIC ULCERATION OF THE TONGUE.

Without going fully into its history, it may be observed that this affection sometimes accompanies rupia, ecthyma, or other forms of cachectic eruption or ulcer, and sometimes occurs independently of any other symptom. Whichever is the case, the patient, in most instances, is palpably below par in constitutional vigour, and, not unfrequently, has suffered from the effects of mercury. He is mostly pallid, and the digestive organs are, in many instances, deranged.

Sometimes the free extremity, most commonly one side of the organ is affected; the median line then, forming a barrier against its extension to the other. There is swelling, sometimes tubercular in form, and hard, or rather boggy to the feel —with ulceration, irregular in shape, and of dirty yellow colour, with picked, or imperfectly healing edges. The ulcers may be deep and fissured, or scooped, or almost superficial. It is not uncommon for a tubercular nodule to ulcerate, and disclose its substance permeated by a yellowish substance, which forms the basis of the ulcer. The analogy between this, and the cachectic subcutaneous tubercle of the skin, can scarcely fail to be perceived.

These ulcers may or may not be painful, they are, at all events, a source of inconvenience, interfering, more or less, both with speech and deglutition.

Slow in formation, tedious in progress, they in many instances last for a very long period, and, in most, are tardy in their disappearance. I have known them heal almost miraculously in a very short space of time, and I have also known them hang on for months and even years.

The treatment which seems to answer best, is such as is generally applicable to cachexia. Sarsaparilla, the iodide of potassium, the mineral acids with bitters, quina, the ammonio-citrate and the iodide of iron, &c. in partial combination, and succession, combined with attention to the digestive organs, and such regimen and diet as are calculated to give tone to the general health.

The effects which follow the exhibition of the iodide of potassium are occasionally as remarkable in this, as in other forms of cachexia.

A gentleman consulted me three or four years ago, with this condition of tongue. He had laboured under it for five years, and a source of great distress it had been to him. He had employed a variety of remedies, and its origin could be traced, in my opinion, to abuse of mercury. I prescribed sarsaparilla and the iodide of potassium, with a suitable course of living, and directed the occasional application of a solution of the nitrate of silver to the fissures, with the daily use of the honey of borax. This gentleman went back into the country, from which he had come to consult me, and I heard no more of him for many months, when he called on me to report the rapid cure which the remedies ordered had effected. In the course of six weeks from commencing them, the tongue appeared quite well. He has since had a slight relapse, but it was quickly arrested by the same measures.

The combinaion of the ammonio-citrate of iron with the iodide of potassium has seemed, on more than one occasion, a highly serviceable one. The formula is this: ℞. Potassii iodidi
Potassæ bicarb.
Ferri-ammonio-cit. sing. ʒss.
Tinct. aurantii c. ℥ss.
Aquæ dist. ℥vss.

M. Capt. æger coch. ij magna bis quotidie, superbibendo (post semi-horam.) Extracti sarsæ concent. c. ℥ss. ex aquæ poculo.

In cachectic ulcerations of the skin, resulting from abuse of mercury, this combination is at times peculiarly valuable.

A gentleman came to me, three years ago, from Lynn, with such ulceration in a severe form upon the leg. It had existed for four or five years, and he had exhausted the medical advice within his reach. He had taken an excessive quantity of mercury, to which, with justice, he attributed the ulceration, which was of serpiginous character, and commenced as soft tubercle. It had not been limited to the limb, but had left unhealthy cicatrices on various parts of the body.

I prescribed the iodine, with iron and sarsaparilla, in the manner above stated, and the result was surprising. In a very few weeks the ulcer healed, the general health was restored, and there has not since been any return of the disease.

I might mention several cases, of a similar description, but it would be tiresome and needless, the experience of other surgeons being capable, no doubt, of supplying many such. Yet it is not always that the issue is so flattering, these cachectic ulcerations, whether of the tongue or of the tegument, being, in too many instances, uncertain, tedious, and obstinate. Cunctando vincere must be our motto, and it is necessary to wear *them* out, and build *the patient's constitution* up, by ringing the changes on tonics and tonic alteratives, to the exclusion, most religiously, of mercury.

Some apology is due for these desultory observations, but their clinical character may excuse their being found in a mere Report of Cases.

### IV. PSORIASIS OF THE TONGUE.

This frequently coincides with the exanthematous mercurial or venereal eruption, with psoriasis guttata of the same character, with condylomatous ulcers of the genitals, which may, however, have only preceded it and disappeared—and, it not unfrequently, exists or survives as an isolated symptom.

The appearance of the tongue varies, in no important degree, from some such standard as this. It has a patchy aspect—spots of the circumference of a pea, or much larger, are red, smooth, shiny, and evidently denuded of epithelium, or having the latter much attenuated. In other parts, there are opaque white patches, varying in number, disposed to be circular, and looking not unlike portions of wax, which had dropped on the tongue in a melted state, and coagulated. The tongue itself is generally sore, sometimes fissured, particularly in the median line, and occasionally swollen, indented by the teeth, reddish, irritable-looking and inflamed. But this latter condition is comparatively rare.

There may, withal, be whitish ulceration of the throat, such as I have described in connexion with ulcerated condyloma,*—or a similarly opaque condition of the epithelium of the palate, and, there may be the several cutaneous complications which I have already mentioned.

---

* Vide Medico-Chirurgical Review—Cyclopædia of Practical Surgery, Art. *Condyloma.*

The patient's health is rarely *bad,* is sometimes excellent, but is commonly indifferent. The previous history of the case varies. In the majority of instances, as I apprehend, it will be found that he has taken a good deal of mercury, and a mercurial affection I believe it, in most cases, to be. In others, we shall find a precursory sore, or condylomata, and nothing which can justify the suspicion of excess of mercury.

The affection, whatever its cause may have been, is too apt to be protracted. The patient gets better, seems well, and is attacked again and again. This, I would say, is peculiarly the case, where a mercurial cause is assignable, and I make a point of warning him of the probable tediousness of his disorder.

The treatment must be materially regulated by the history. If there has already been much mercury taken, I would most earnestly advise that no more should be given.

Sarsaparilla and the iodide of potassium appear to be, on the whole, the most efficient remedies, but change is as necessary for this as for the cachectic affection, and the object to be kept steadily in view, whatever measures are employed, is to sustain the general health. The ammonio-citrate or the iodide of iron I have found very serviceable here.

As a local application, none is comparable to the lunar caustic. The opaque spots upon the tongue may be touched with it twice or three times in the week. If the palate is affected it may be treated in the same manner, or painted with a strong solution of the same salt. The gargle of the bichloride of mercury, one grain to six ounces of water, or that of the sulphate of zinc, have their merits, and, in fact, many slightly stimulating applications are of use.

As an instance of the bad consequences of much mercury in this affection, I may advert to the following case.

It is some seven or eight years since I first saw the gentleman, the subject of it. He had then "psoriasis" of the tongue, and an exanthematous efflorescence pretty generally on the body. He was about 22 years of age, stout, robust, and with every evidence of a good constitution. He stated that about two years previously, while reading in the long vacation, (he was studying at Oxford,) he contracted what he supposed was a venereal sore. The gentleman whom he consulted assured him it was not, and for two months, while he continued under his care, he took no mercury whatever. At the end of that time he went to Oxford. The sore was then healed, but the cicatrix was extremely hard. The surgeon whom he consulted prescribed twenty-five grains of blue-pill daily, which he continued to take for seven weeks. The hardness of the cicatrix disappeared, his mouth was not made sore, and he appeared to be cured. But soon the throat grew sore, and a rash broke out upon the skin. For this he had advice in town, and again took mercury along with sarsaparilla. Prior to my seeing him he had had several such courses by the recommendation of various medical men. The rash would disappear for a time, and without apparent cause return.

I endeavoured to dissuade him from resorting to further mercurial treatment, and prescribed sarsaparilla, the mineral acids, and so forth. But my patient was difficult to be controlled, and indulged in excesses, more particularly in drinking, against which cautions were vain. He disregarded, also, my injunctions with respect to mercury, and, with the sanction of others, underwent one or two courses more. Yet, after two or three years spent thus, he seemed, at last, to be well, and no further eruption occurred on the skin, nor did aught seem amiss with the tongue or throat. So he continued for two or three years, when, without any warning, one lachrymal bone grew carious. Ulcerations of the Schneiderian membrane followed, and the bony septum of the nose in its anterior part was destroyed. Then came caries and exfoliation of the palatine plate of the palate-bone, and the disease is still going on in the nose, without any prospect of speedy or permanent recovery.

It would be difficult, I conceive, to exonerate mercury from the blame of what happened in this case, though the patient's own excesses in other ways, contributed to aggravate the mischief.

### V. SMALL SCIRRHOUS-LIKE TUMOR OF THE FRÆNUM LINGUÆ IN A CHILD.

Frederick Pigott, aged 13 months, was brought to me by his mother, on account of a tumor beneath his tongue, interfering with articulation and with eating.

The tumor occupied the frænum, was attached to, or rather blended with, the gum below, and reached to the tongue, with which it was *not* blended, above. It was bigger than a horse-bean, of irregular shape, ulcerated upon the superficies with everted edges, hard to the feel, and in appearance not very unlike scirrhus. But the child was healthy, and there was no contamination of the lymphatic glands.

The mother had noticed it two months previously, just before he cut his central incisors. It began as a white speck, which had gradually increased up to its described dimensions.

In the course of a few days, I removed the tumor with the ligature, this being preferable to the knife on account of the ranine vessels. The only difficulty was occasioned by the young patient's refractoriness, but, by drawing out the tumor with a hook, the needle armed with a double ligature was passed, and the diseased part included in it. The slough separated; for security's sake, nitric acid was applied to the wound; this healed; and the child appears to be cured.

A case of RANULA presented itself. The patient was a girl, aged 13, the tumor was as large as a bantam's egg, of bluish colour, and evidently fluctuating. There was little impediment to speech or deglutition. The disease had existed for three months.

I passed a seton cross-ways through the ranula, and a quantity of glairy fluid escaped, the swelling subsiding much in consequence. I regret that I cannot report the result, the patient, soon afterwards, ceasing to attend.

### VI. PUNCTURE SUCCEEDED BY BLISTERS FOR COLLECTION OF FLUID IN SUBCUTANEOUS BURSÆ.

In several cases of enlargement of the supra-patellar and supra-olecranal bursæ, from fluid connected within them, I have lately adopted the plan of first puncturing the sac with a grooved needle, and allowing the fluid to escape, and then applying blisters. The fluid forms again in the bursa, but is speedily absorbed, and it appears that time is gained by the proceeding. The two cases which follow are taken at hazard as samples.

*Case* 1.—Catherine New, aged 23, housemaid, applied July 12, 1843.

Right bursa patellæ as large as a duck's egg—sac thin—skin red and much distended—little inflammation. Pulse small and weak, health rather delicate.

Complaint has existed for fourteen months.

*Puncture with grooved needle—much fluid let out.* Emplast. lyttæ genu.

15th. No inflammation. Sac nearly filled. Blister just healed.

Rep. emp. lyttæ.

22nd. Effusion nearly gone.

Rep. emp. lyttæ.

After this there was no return of effusion, and some slight thickening of the sac seemed to occur. A bandage and strapping were applied, and on the 5th of August, she was discharged cured.

*Case* 2.—Ann Neales, aged 22, servant, applied August 16, 1843.

Effusion into right bursa patellæ, of two months standing. Bursa of the size of a small egg. No inflammation.

*Punctured.* Emplast. lyttæ.

The effusion returned in a less degree, the blister continued open, and it was not before the 1st of September that a second was applied. The effusion was then gone, and this was only for precaution's sake.

On the 9th the knee was bandaged, and she was discharged cured.

Of the permanence of the cure in these cases, I have no experience. In some, I dare say, it will, and in some will not, succeed. For the patients are usually compelled to resume their laborious kneeling, which as it induced, may naturally be expected to restore the complaint. But this seems to me an improvement on the plan of *mere* blistering, the one generally resorted to.

In *subcutaneous* bursæ, it cannot be hazardous, indeed I usually move the needle about, for the purpose of exciting inflammation. This, as in the case of hydrocele, may lead to adhesion of the walls of the sac, or tend to arrest the secretion of fluid in it.

### VII. DIFFUSE INFLAMMATION OF THE DEEP CELLULAR MEMBRANE OF THE PELVIS.

The affection to which I would direct attention is not, I believe, very generally understood, at least in connexion with such cases as I shall allude to. The present notice is not intended to supply the deficiency, but rather to give a hint to surgeons, and to precede some more extended observations of my own.

The point to which I would advert at present, is the risk of inflammation of the cellular membrane of the pelvis from operations for hæmorrhoids or fistula, or even independently of any operation whatsoever. The general character and history of the affection may be briefly sketched as follows:—

Two or three days after the pile has been tied, or the sinus has been laid open, a rigor, more or less distinct, ushers in pyrexia, with its usual features, heat of skin, frequent pulse, thirst, perspirations, and impaired secretions. But the symptoms are of rather a low character, and there is an expression of anxiety, exceeding their apparent gravity. Along with the feverishness, there is pain complained of in the lower part of the abdomen, and more particularly, I would say, in the groin. This has seemed to me an early symptom, and it is always a suspicious one. All this while, there may be little to attract attention about the rectum or the wound, the latter, if any thing, exhibiting a tendency to dryness.

With the progress of the disorder, the rigors may or may not return, there may or may not be sickness, but there *is* a frequency of pulse which never fails,* and an anxiety of countenance which is never wanting. Some pain, too, persists in the belly. It is not limited to one spot—perhaps it may be greatest in the groin, but it may even reach to the diaphragm. Not, however, that the patient will complain very much of it—he may rather be disposed to make light of it.

Possibly, the inguinal glands may enlarge—they may even have done so at the first—the belly grows tympanitic—there is a disposition to sweats—perhaps

* In my experience, a pulse *remaining* frequent after injuries or operations, is always a dangerous, though not, of necessity, a fatal symptom.

rigors alternate with them. So the case may go on for some days, a week, or even more. Then may come hurried respiration, slight cough, obscure pain in the thorax, low delirium, death. The patient is cut off by secondary pleuro-pneumonia or deposits.

Or the termination may be more sudden. With the tympanitis the depression increases, the tongue embrowns, there is delirium, and the patient dies unexpectedly.

The subject of such symptoms is commonly cachectic, one broken down by mercury or by spirit-drinking, or by some depressing agency. But, in other instances we have not cachexia, but a highly nervous temperament to deal with—a hysterical female, rather spent in years, or a man whose mind has been severely taxed.

When we come to examine the body after death, we find (this is not necessary) some traces of recent peritoneal inflammation. It is not "adhesive inflammation" that we see—not lymph and agglutination of the coils of bowel; but diffused vascularity, and turbid serum, with dirty pus dissolved in it, or flocculi of lymph, and when such appearances are found, they are principally about the pelvis.

I said they were not necessary, nor are they. There may not exist the faintest trace of peritoneal inflammation. Be it there or not, it does not constitute the gist of the disease. That is in the sub-peritoneal cellular tissue. In that we find pus or concrete lymph, diffused perhaps to a great extent. We commence discovering it in the vicinity of the tied pile or divided sinus—thence it spreads along the rectum to the pelvis, behind the mesentery to the belly, up to the kidneys, to the diaphragm. It does not, of necessity, go so far, that hinges on the case, but it is always found, and it palpably has its source about the rectum. It is, in short, diffuse inflammation and suppuration in the cellular membrane of the pelvis, spreading by continuity to the sub-peritoneal cellular tissue, and affecting by contiguity the serous membrane. That secondary inflammations of the pleura or the lung, or deposits any where, should under such circumstances occur, is no more than might naturally be expected.

On the treatment of this affection I have little to say. Whenever the symptoms that bode it, have appeared, we must *dread* the worst result. Not that I believe this always follows; indeed I have little doubt that it does not. But it is very apt to follow, and the surgeon ought to be prepared for it. Incisions, our main hope in diffuse cellular inflammation elsewhere, are here out of the question. The mischief is beyond the knife. Moderate local depletion may be serviceable, active depletion is not. Support must probably go along with measures, calculated, like leeches and blisters, to keep down local action. The diffused character of the inflammation and effusion forbids on the one hand lowering remedies, and gives little encouragement to anticipate benefit from any.

Perhaps, the best lesson we can draw from an acquaintance with the affection is this—to be chary of operating on such persons as likely subjects for it—and to sustain, by diet and by other means, those on whom we do operate.

